# CGRP Plasma Levels Correlate with the Clinical Evolution and Prognosis of Hospitalized Acute COVID-19 Patients

**DOI:** 10.3390/v14102123

**Published:** 2022-09-26

**Authors:** Manuela Rizzi, Stelvio Tonello, Francesca Morani, Eleonora Rizzi, Giuseppe Francesco Casciaro, Erica Matino, Martina Costanzo, Erika Zecca, Alessandro Croce, Anita Pedrinelli, Veronica Vassia, Raffaella Landi, Venkata Ramana Mallela, Davide D’Onghia, Rosalba Minisini, Mattia Bellan, Luigi Mario Castello, Francesco Gavelli, Gian Carlo Avanzi, Filippo Patrucco, Mario Pirisi, Donato Colangelo, Pier Paolo Sainaghi

**Affiliations:** 1 Department of Translational Medicine, Università Del Piemonte Orientale (UPO), 28100 Novara, Italy; 2 CAAD, Center for Autoimmune and Allergic Diseases, Università Del Piemonte Orientale (UPO), 28100 Novara, Italy; 3 Department of Internal Medicine and COVID-19 Unit, AOU “Maggiore Della Carità”, 28100 Novara, Italy; 4 Division of Emergency Medicine and COVID-19 Sub-Intensive Unit, AOU “Maggiore Della Carità”, 28100 Novara, Italy; 5 Internal Medicine and Rheumatology Unit, AOU “Maggiore Della Carità”, 28100 Novara, Italy; 6 Division of Internal Medicine, Azienda Ospedaliera “SS. Antonio e Biagio e Cesare Arrigo”, 15121 Alessandria, Italy; 7 Medical Department, Division of Respiratory Diseases, AOU “Maggiore Della Carità”, 28100 Novara, Italy; 8 Department of Health Sciences, Pharmacology Unit, Università Del Piemonte Orientale (UPO), 28100 Novara, Italy

**Keywords:** calcitonin gene-related peptide (CGRP), COVID-19, pulmonary intravascular coagulopathy, IP10

## Abstract

SARS-CoV-2 is the etiological agent of COVID-19, an extremely heterogenous disease that can cause severe respiratory failure and critical illness. To date, reliable biomarkers allowing for early patient stratification according to disease severity are still lacking. Calcitonin gene-related peptide (CGRP) is a vasoactive neuropeptide involved in lung pathophysiology and immune modulation and is poorly investigated in the COVID-19 context. In this observational, prospective cohort study, we investigated the correlation between CGRP and clinical disease evolution in hospitalized moderate to severe COVID-19 patients. Between January and May 2021 (Italian third pandemic wave), 135 consecutive SARS-CoV-2 patients were diagnosed as being eligible for the study. Plasma CGRP level evaluation and routine laboratory tests were performed on blood samples collected at baseline and after 7 days of hospitalization. At baseline, the majority our patients had a moderate to severe clinical presentation, and higher plasma CGRP levels predicted a higher risk of in-hospital negative evolution (odds-ratio OR 2.84 [IQR 1.07–7.51]) and were correlated with pulmonary intravascular coagulopathy (OR 2.92 [IQR 1.19–7.17]). Finally, plasma CGRP levels were also correlated with plasma IP10 levels. Our data support a possible crosstalk between the lung and the neuroimmune axis, highlighting a crucial role for plasma CGRP in sustaining COVID-19-related hyperinflammation.

## 1. Introduction

Even if the last two years have been important for the comprehension of the crucial common mechanisms driving the immune response and the inflammation process, the COVID-19 pathophysiology remains unclear to some extent [1,2,3]. The heterogeneous clinical presentation of patients to SARS-CoV-2 infection and the different response to treatment regimens limit the personalization of drug protocols. Some patients develop a hyperinflammatory reaction to the infection, the so-called cytokine storm, but little information is available about adopting surely effective approaches to prevent it [4]. Although many inflammatory mediators or vasoactive peptides (interleukin (IL)-6, IL-8, tumor necrosis factor alpha (TNF-α), angiotensin II (Ang II), vasoactive intestinal peptide (VIP), and endothelin 1 (ET-1)) have been proposed as biomarkers of disease evolution, so far, it is difficult to have a clear prognosis for patients or indications in order for a more proper pharmacological strategy to be promptly adopted [5].

Among these markers, calcitonin gene-related peptide (CGRP) is a suitable candidate because of its implication in immune system function and inflammatory processes [6,7,8]. In fact, CGRP participates in neurogenic inflammation, in the regulation of vascular plasticity and reactivity, in the modulation of the release of inflammatory cytokines (including IL-6), in microglia activation, in sepsis and septic shock inflammatory cascades, in airways through hyperemia and capillary permeability, and in adaptive immune responses [8]. To date, clinical and experimental data indicate that CGRP might be responsible for the initiation of the inflammatory process, but, on the other hand, they seem to also have some anti-inflammatory properties, for example by inducing IL-10, and, more interestingly, in the tissue and endothelium damage repair response, especially after ischemic insult [9,10,11,12,13,14]. Furthermore, it has been reported that the CGRP receptor complex cooperates in bronchial protection, and that the expression of its subunit, the receptor activity modifying protein 1 (RAMP1), is high in lung samples [15,16]. It is noteworthy that this receptor is expressed in dendritic cells, macrophages and human CD34+ cells, hematopoietic progenitor cells, and B and T lymphocytes, and thus the role of CGRP might be both addressed as inflammation trigger and post-inflammation damage repair inducer. The interpretation of the role of CGRP in these processes is complicated by the rapid desensitization and degradation of the receptor complex after a sustained stimulation [16].

Consistently, the adoption of CGRP antagonists, which are new pharmacologic agents useful in hemicrania prophylaxis, in COVID-19 treatment has not been fully evaluated yet [17].

To fill these gaps, we evaluated a selected cohort of 135 patients affected by COVID-19 pneumonia and respiratory failure eligible for admission in high dependency/sub-intensive units. We measured the CGRP plasma levels upon admission and after 7 days of hospitalization, and analyzed the correlation of these levels with the clinical evolution.

## 2. Materials and Methods

### 2.1. Patients

Here, 139 consecutive patients hospitalized in non-intensive care unit (non-ICU) wards (including high dependency/sub-intensive units) of “Maggiore della Carità” University Hospital in Novara (Italy) between January and May 2021 (corresponding to the third pandemic wave in Italy) were asked to participate in a clinical study that aimed to identify prognostic biomarkers in COVID-19 patients. This study is a satellite of a larger multicenter observational study project (BIAS—Baseline Immunity status effect on SARS-CoV presentation and evolution: comparison between immunocompetent and immunocompromised patientS study), conducted in strict accordance with the Declaration of Helsinki. As human subjects were involved, the study protocol was approved by the local Ethical Committee (CE 7/21) and all of the enrolled patients were asked to sign an informed consent form. To be eligible for this study, patients needed to meet the following inclusion criteria: being adults (>18 years), needing hospitalization due to SARS-CoV-2 infection (positivity was assessed either by RT-PCR or third generation antigenic tests), and with clinical symptoms not exceeding 12 days. Patients with a very severe clinical presentation, suggestive of an imminent death or of an immediate ICU admission, as well as patients with advanced cancer (i.e., not suitable for medical or surgical treatment) or stage V renal failure (glomerular filtration rate < 15 mL/min) were excluded [18].

All the enrolled patients received a standard of care treatment upon admission, as defined by the “Maggiore della Carità” University Hospital internal guidelines for COVID-19 patient management. These patients received oxygen supplementation, corticosteroids, and low molecular weight heparin (LMWH) when appropriate, unless contraindicated.

### 2.2. Endpoints Definition

The predefined primary endpoint was defined as the correlation of plasma CGRP levels, assayed at baseline and after 7 days of hospitalization, with disease evolution, defined as unfavorable (in-hospital death or ICU admission) or with rapid clinical recovery (discharge and/or stable National Early Warning Score 2 (NEWS2) ≤ 2 for at least 24 h within the first 14 days of hospitalization). Secondary endpoints were defined as the correlation between plasma CGRP levels and some clinical features, such as the occurrence or high suspicion of pulmonary intravascular coagulopathy (defined as a diagnosis of pulmonary embolism confirmed with contrast-enhanced chest CT scan or elevation of D-dimer above normal reference values after age correction associated to clinical deterioration after admission), the occurrence of cardiac complications, blood pressure alterations, headache, and delirium [19,20].

### 2.3. Blood Sample Collection

Blood samples for routine hematological evaluation, as well as for CGRP quantification, were collected by venous puncture using EDTA as the anticoagulant at different time points along hospitalization (at the time of hospital admission (baseline, t0) and after 7 days of hospitalization (t7)). Blood fractions were immediately separated by centrifugation and stored at −80 °C until the time of the analysis.

### 2.4. Routine Laboratory Evaluation

Blood samples from each patient were analyzed in clinical practice to obtain a complete cell count, a common biochemistry (i.e., aspartate aminotransferase—AST; alanine aminotransferase—ALT; creatinine), inflammatory (i.e., C reactive protein—CRP; ferritin) and coagulation/fibrinolysis (i.e., D-dimer) panel.

### 2.5. CGRP Quantification

The plasma CGRP levels were determined by the ELISA technique using a commercial kit (MyBioSource Inc, MBS267126, San Diego, CA, USA) following the manufacturer’s instructions [15]. Prior to CGRP quantification, samples were assessed to identify the correct dilution (1:25 in dilution reagent, provided by the manufacturer). Absorbance was recorded using a Victor X4 microplate reader (Perkin Elmer, Waltham, MA, USA). Optical density at 450 nm was fitted versus a calibration curve prepared with CGRP standard (range 0–1000 pg/mL), as suggested by the manufacturer.

### 2.6. Multiplex Analyses

Here, 27 plasma cytokines, chemokines, and growth factors were analyzed using the commercial Bio-Plex Pro Human Cytokine 27-plex panel (Bio-Rad Laboratories Inc., Hercules, CA, USA), as previously described [18].

### 2.7. Data Collection

Demographics, clinical parameters, therapeutic schedule, and laboratory parameters for each selected patient were stored and managed on a web-based encrypted database (REDCap platform) [21]. Relevant data were collected by reviewing medical records, starting from hospital admission (t0, baseline) until study exit (achievement of either the positive or negative endpoint or up to a maximum of 28 days).

### 2.8. Statistical Analyses

Data extracted from the REDCap database and CGRP quantifications were analyzed to evaluate their statistical significance toward the previously described endpoints. Continuous variables were expressed in terms of measures of central tendency and dispersion (medians and interquartile range (IQR)), while categorical variables were expressed as frequencies (percentages). Statistical analyses were based on Mann–Whitney U test (for continuous variables). Statistically significant values identified by univariate analysis were used to build multivariable stepwise regression models. Laboratory data collected in clinical practice and for research purposes were also used for a multiple correlation analysis. Furthermore, we also built ROC (receiver operator characteristics) curves to identify the prognostic cut-off for the parameters of interest. The statistically significant threshold was set at 0.05 (two-tailed). Statistical analyses were performed with Statistica for Windows release 12 (TIBCO Software Inc, Palo Alto, CA, USA) and MedCalc^®^ Statistical Software version 20.014 (MedCalc Software Ltd., Ostend, Belgium).

## 3. Results

Between January and May 2021, during the third Italian pandemic wave, we enrolled and prospectively followed 139 patients admitted to non-ICU wards of “Maggiore della Carità” University Hospital, Novara, Italy, for moderate or severe COVID-19, with the aim to identify novel prognostic biomarkers [18,22,23]. Out of the initial 139 patients, 135 were found to be eligible for plasma CGRP evaluation, while 4 patients were excluded due to the lack of the corresponding plasma sample. The median age of the enrolled patients was of 63.8 (IQR: 56–72) years and, as also expected by the available literature on severe COVID-19 clinical evolution, many of them were represented by male subjects (61.5%). Detailed demographical and baseline (t0) clinical description, as well as the most common symptoms at hospital admission for the study cohort, are shown in Table 1 and Table 2.

At the time of hospital admission, 74.1% of the enrolled patients showed moderate respiratory failure (100 ≤ PiO_2_/FiO_2_ < 200), while 6.7% had a severe clinical presentation (PiO_2_/FiO_2_ < 100). Moreover, before hospital admission, many patients were already receiving a COVID-19-related home treatment based on corticosteroids (53.3%), azithromycin (35.6%), and heparin (31.1%), alone or in combination.

As disease severity upon admission was relatively high, as confirmed by the baseline NEWS2 score of 5 (IQR:4–6) [24], all hospitalized patients with moderate to severe symptoms underwent a standard therapy based on oxygen supplementation, corticosteroids (dexamethasone (8 mg/die) or methylprednisolone (80 mg/die), if displaying a PiO_2_/FiO_2_ ratio lower than 200), and LMWH (enoxaparin 4000 U.I./die or 100 U.I./kg twice a day, according to thrombosis clinical suspect), unless contraindicated.

Among the 135 patients included in the CGRP cohort, 29 (21.48%) had a negative outcome (in-hospital death or ICU admission), while 87 (64.44%) reached the positive endpoint (discharge and/or NEWS2 ≤ 2 for at least 24 h within 14 days of hospitalization).

Table 3 and Table 4 show that baseline plasma CGRP levels were correlated with the patient clinical evolution. The baseline plasma CGRP concentration was significantly higher in patients with a negative evolution (death or ICU admission) (Table 3) with respect to all other patients (1.02 ng/mL vs 0.91 ng/mL, *p* = 0.05). Consistently, the baseline plasma CGRP concentration was lower in patients who had a faster clinical recovery (discharge and/or NEWS2 ≤ 2 for at least 24 h within 14 days of hospitalization) with respect to all other subjects (0.90 ng/mL vs 0.97 ng/mL, *p* = 0.02; Table 4). There were no differences in the plasma CGRP concentration when measured at 7 days from hospital admission when performing the comparisons as above (Table 3 and Table 4).

Considering the secondary endpoints, we observed a statistically significant correlation between baseline plasma CGRP levels, and the development of pulmonary intravascular coagulopathy (Table 5). In those patients, we observed a higher baseline plasma CGRP level compared with those that did not develop pulmonary intravascular complications (0.99 ng/mL vs 0.88 ng/mL, *p* < 0.01).

No statistical correlation between baseline or 7 days for the plasma CGRP levels and the other predefined secondary endpoints, such as headache, delirium, cardiac complications, and blood pressure variations, were observed.

For the multivariate analysis, baseline plasma CGRP levels retained their prognostic role towards the negative (in-hospital death or ICU admission) and positive (discharge and/or NEWS2 ≤ 2 for at least 24 h within 14 days of hospitalization) endpoints, after correction for demographic variables such as age and gender, and for disease severity parameters such as PiO_2_/FiO_2_ and NEWS2, as shown in Table 6 and Table 7.

Considering pulmonary vascular complications, during the multivariate analysis, the baseline plasma CGRP concentration retained its prognostic role towards the endpoint, even after the correction for demographic and disease severity variables, as shown in Table 8.

We further investigated if there was any possible correlation between CGRP plasma levels at baseline (t0) and other laboratory parameters related to clinical severity or inflammation. As shown in Table 9, the only significant correlation was observed with IP10, the interferon-γ-inducible protein 10 (IP10/CXCL10). This protein is known to be involved in the initiation and progression of infectious diseases and its transient early surge significantly correlates with SARS-CoV-2 viral load in mild patients [25].

**Figure 1:**
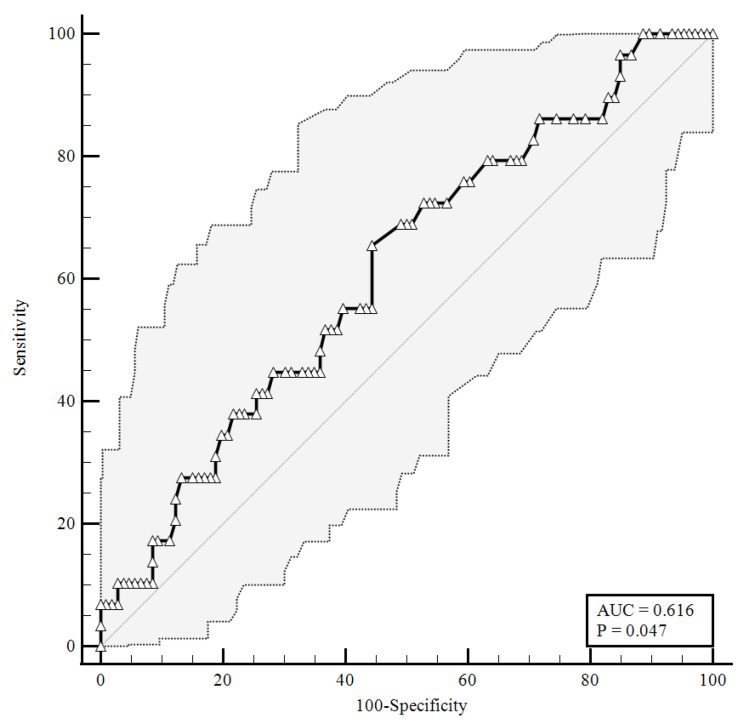
ROC curve for plasma CGRP levels at the time of hospital admission predicting a severe disease evolution (65.52% sensitivity, 55.66% specificity). AUC = area under the curve, *p* = *p*-value.

**Figure 2:**
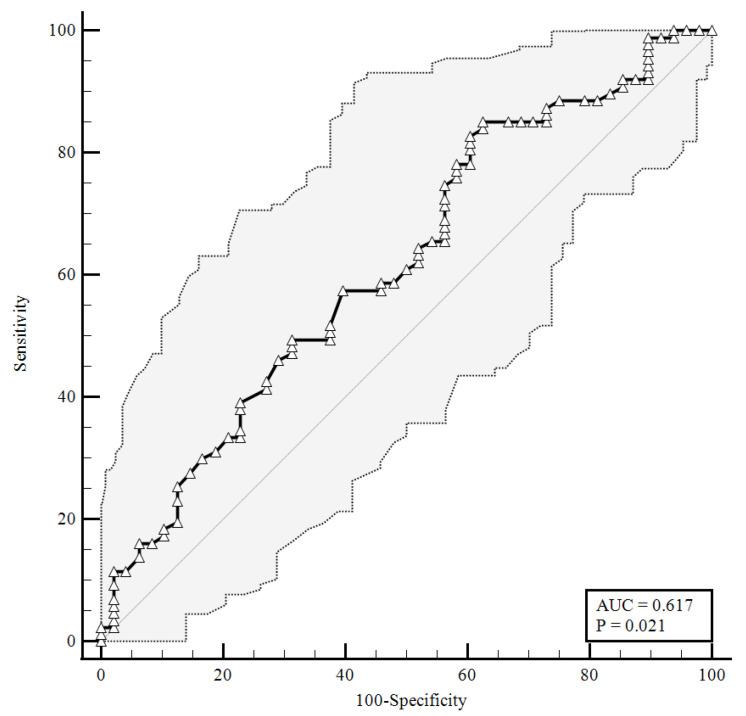
ROC curve for plasma CGRP levels at the time of hospital admission predicting a faster clinical recovery (85.06% sensitivity, 37.5% specificity). AUC = area under the curve, *p* = *p*-value.

**Figure 3:**
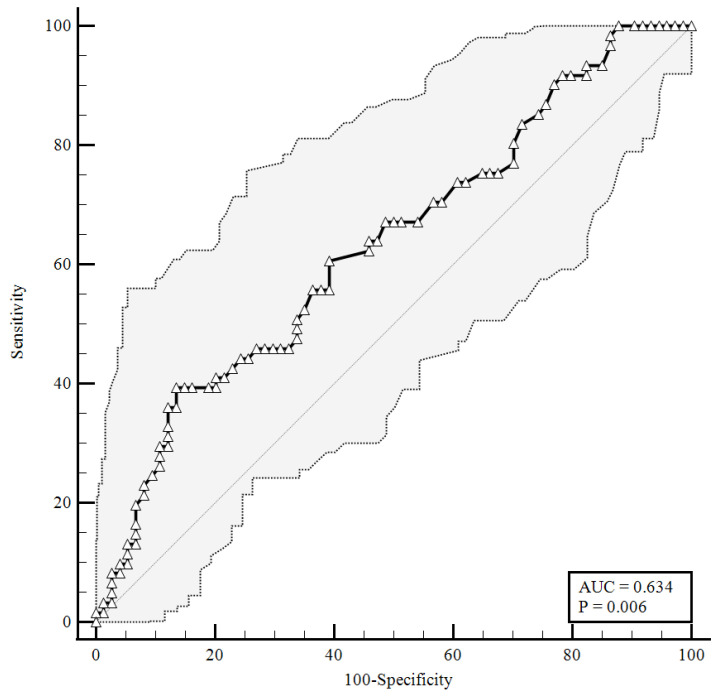
ROC curve for plasma CGRP levels upon hospital admission predicting pulmonary vascular events development. AUC = area under the curve, *p* = *p*-value.

To identify the prognostic cut-offs, we built ROC curves for baseline plasma CGRP values, which referred to the results presented above. For the ROC analyses, sensitivity is defined as “positivity in disease”, and refers to the proportion of subjects who have the target condition (true positives), while specificity is defined as “negativity in health” and refers to the proportion of subjects without the target condition (true negatives) [26]. In our simulation, we defined three target conditions, severe disease evolution (Figure 1), faster clinical recovery (Figure 2), and pulmonary intravascular coagulopathy (Figure 3).

As shown in Figure 1, baseline plasma CGRP levels higher than 0.92 ng/mL were predictive of a negative disease evolution (area under the curve (AUC) = 0.616, 65.52% sensitivity, 55.66% specificity), with a likelihood ratio of 1.48 (95% confidence interval (95%CI): 1.05–2.07).

As shown in Figure 2, a baseline plasma CGRP level lower than 1.26 ng/mL (AUC = 0.617) predicted a faster clinical recovery (85.06% sensitivity and 37.5% specificity), with a likelihood ratio of 1.36 (95% CI: 1.07–1.72).

As shown in Figure 3, considering an area under the curve of 0.634, the plasma levels of the CGRP at a baseline higher than were 1.23 ng/mL correlated with pulmonary intravascular coagulopathy with 39.34% sensitivity and 86.49% specificity, and a likelihood ratio of 2.91 (95% CI: 1.51–5.61).

## 4. Discussion

Our data offer the possibility to study a specific population of COVID-19 patients admitted to high dependency/sub-intensive units due to the severity of their clinical status. Standard pharmacological treatment was administered to these patients according to the current guidelines for hospitalization. Of our cohort, 29 out of 135 patients had a negative disease evolution requiring ICU admission or leading to in-hospital death. On the other hand, 87 out of 135 patients had a faster clinical recovery, with discharge or stable NEWS2 ≤ 2 within 14 days. Considering this, the identification of a biomarker able to predict clinical evolution and to direct therapeutic decisions from the admission to high dependency/sub-intensive units, would be of great clinical interest. To date, there are no clear indications to guide a differential approach at admission. The aim of this study was to identify a biomarker to help clinicians with identifying, upon admission, the differences between patients to be used for stratifying the risk of disease progression and to have indications for a more appropriate pharmacological regimen. Our results seem to indicate a possible role for the baseline plasma CGRP level at predicting, upon admission, a severe disease evolution and to recognize patients with a better prognosis. In fact, a low level of this peptide predicts, with a high sensitivity (85.06%), a faster clinical recovery, and its high specificity allows for recognizing patients that are not able to recover within 14 days (cut-off < 1.26 ng/mL). It is out of note that the predictive value of this peptide toward a negative disease evolution was also retained when the data were corrected for age and gender (Table 6), further confirming the current knowledge about the higher vulnerability of older males (>60 years old). Additionally, the assessment of the plasma CGRP concentration seemed to offer a wide range of clinical information. As shown in Figure 3, this determination might also be used to assess the presence of pulmonary intravascular coagulopathy (cut-off > 1.23 ng/mL).

As the increase in plasma CGRP levels participates in triggering inflammation and vascular reactivity, it is conceivable to suppose that patients admitted to high dependence/sub-intensive wards with high levels of this bioactive peptide already experienced and/or are experiencing pulmonary and vascular events. Thus, in our opinion, plasma CGRP levels seem to be more indicative of a sub-cohort of patients that have already manifested some clinical features of disease progression. This stratification could be useful to suggest the need for aggressive therapeutic approaches.

The population that we considered possessed homogeneous characteristics upon admission, thus giving us the possibility to have a simpler framework to interpret the clinical meaning of plasma CGRP quantification. It might be assumed that circulating CGRP levels, if applied in the follow up of SARS-CoV-2 positive individuals, might help in detecting early pulmonary intravascular coagulopathy, which predispose to the adverse progression of the disease. In this view, CGRP evaluation might also be applied to other severe clinical conditions involving hyperinflammation or dysregulation of the immune response.

D-dimer is associated with pulmonary intravascular coagulopathy in COVID-19 patients and is more frequently indicative of microthrombosis rather than macrothrombosis (responsible of most hospitalized COVID-19 acute respiratory distress syndrome) [20,27]. The most recent NIH COVID-19 Treatment Guidelines already consider D-dimer elevation as a potential marker for anticoagulative therapy in low or intermediate risk hospitalized patients [28]. Some authors have described that D-dimer median values are different in mild or severe COVID-19 patients. A marked increase has been described in patients with severe COVID-19, and meta-analyses confirmed that values of D-dimer greater than 500 μg/L were descriptive of severe disease [28,29]. These considerations support our definition of suspected pulmonary coagulopathy in our cohort.

Even if CGRP involvement in lung physiology has been extensively studied [5,30,31,32], its potential role in COVID-19 is poorly understood. To date, only a few studies have focused on this topic [15,33], with no possibility of obtaining conclusive results. Bolay and coworkers investigated different circulating inflammatory biomarkers in 88 COVID-19 patients with and without associated headache. For this study, they enrolled patients with moderate disease, hospitalized in regular wards, during a two-month timeframe. In this context, these researchers evaluated the serum CGRP levels in patients with and without headache and did not find any difference in the biomarker circulating levels within the two groups [33]. Even if these researchers did not give any detailed information about the therapeutic regimen adopted, their data about the serum CGRP levels and headaches support our observations about a lack of correlation between plasma CGRP levels and migraine in moderate to severe hospitalized patients. On the other hand, the Ochoa-Callejero research group showed low serum CGRP levels in COVID-19 patients, along with an increased lung RAMP1 expression, which is supposed to compensate for the decrease in systemic CGRP levels [15]. The major limitations of this work are represented by the limited number of hospitalized patients (23 in the normal wards and 10 in the ICU) and by the lack of any detailed information about the disease manifestations leading to hospital admission and a therapeutic regimen in hospitalized patients. Our results, highlighting a higher level of plasma CGRP in patients with a negative disease evolution, seem to be opposite to that of Ochoa-Callejero and coworkers, but this could be explained by the larger number of moderate to severe patients with a well-defined therapeutic regimen enrolled in our study.

It is known that CGRP is a widely expressed neuropeptides, which can be detected not only in nerve fibers, but also in other non-nervous districts, such as the heart, the respiratory system, and the vasculature, where its receptors could be found in all of the vessel’s cellular layers [34,35]. Focusing on the vascular district, it is interesting to note that endothelial cells not only express CGRP receptors, but are also able to synthesize the peptide, which can be released either in the blood flow or in the subendothelial layer, depending on the vascular microenvironment, thus supporting CGRP involvement in the autoregulation of local hemodynamics [34,36,37,38].

Interestingly, it has been observed that in healthy subjects, the plasma CGRP levels are generally low. The increase is generally associated with pathological conditions, such as sepsis, thus supporting CGRP involvement in immunomodulation [34,39]. The involvement of this neuropeptide in inflammation is supported by the observation that it is synthesized and released following tissue injury, supporting the local infiltration of inflammatory cells and the regulation of antigen presenting cells activity, as observed in different chronic inflammatory diseases [5,8,39]. Furthermore, CGRP has been demonstrated to stimulate IL-6 production [5,6,40]. The increase in IL-6 levels is a classical hallmark of cytokine-storm-related coagulopathy, as this proinflammatory cytokine is known to promote coagulation cascade activation and vascular leakage. The subsequent endothelial dysfunction is essential in sustaining cytokine storm severity as it amplifies the ongoing inflammatory reactions, which result in an increased risk of microvascular thrombosis and respiratory failure [41,42].

CGRP involvement in inflammatory responses through the IL-6 pathway further supports our results, highlighting the correlation between higher peptide levels in plasma and worse disease evolution, as well as with pulmonary intravascular coagulopathy. The complex CGRP physiology accounts for the observed appropriate or detrimental effects of CGRP or CGRP receptor antagonist drugs in different clinical conditions [6,26]. To date, monoclonal antibodies targeting CGRP are available to treat migraines [43] and are safe in clinical practice in terms of COVID-19 infection susceptibility [44,45]. Moreover, it has been hypothesized that in COVID-19, a crosstalk between the lungs and the neuro–immune axis could take place, sustaining the development of a clinical trial aimed to investigate the effectiveness of vazegepant, an anti-CGRP molecule initially developed to treat migraines, in COVID-19 management [45].

We observed an interesting correlation between CGRP and IP10 levels measured upon admission in our non-ICU ward. This was the only correlation between CGRP and was a marker of clinical disease progression and severity that could be demonstrated in these patients. In our opinion, such a correlation strengthens the potential role of CGRP quantification upon admission, merely for a more accurate clinical risk ranking of patients that already manifested some clinical features of the disease, such as possible microthrombosis, as IP10 is mostly linked to hyperinflammation due to monocyte/macrophage hyperactivation rather than endothelial injury or activation [18]. Furthermore, if assessed together with a selected panel of biomarkers, CGRP quantification might play a decisive role in risk assessment and in driving early clinical decisions toward appropriate preventive pharmacological interventions. This statement is in accordance with other authors that have already proposed vasoactive peptide assessment as a routine part of COVID-19 patient monitoring [5].

We are aware that our study has some limitations. First, we focused on non-ICU hospitalized patients with moderate or severe symptoms, so it is not possible to extend these observations to mild or even asymptomatic patients without performing dedicated studies. In addition, the mono-centric nature of this study and the limited number of patients enrolled could represent a limitation and, before proposing circulating CGRP quantification in clinical practice, a prospective multicentric study will be mandatory. Finally, we assumed linear behavior of our data for statistical purposes, making it possible that some confounding factors could have influenced the obtained results.

## 5. Conclusions

To date, no specific marker has been proposed to monitor the disease trajectory or for stratifying SARS-CoV-2 positive patients early on. Our group has already proposed the use of routine non-COVID-19 specific laboratory parameters, such as red cell distribution width (RDW), neutrophil-to-lymphocyte (NL) ratio, and platelet count, for predicting in-hospital mortality [46]. In this work, we propose the evaluation of CGRP to anticipate and describe the pulmonary intravascular coagulopathy that occurred in SARS-CoV-2 positive patients when admitted to high dependency/sub-intensive units. Thus, this peptide represents a new opportunity to early identify COVID-19 patients that might require more incisive clinical monitoring and a more appropriate pharmacological approach.

## Figures and Tables

**Table 1:** Demographic and baseline clinical characteristics of the study population cohort. § refers to data obtained with oxygen supplementation. IQR = interquartile range.

Demographics, Parameters, and Clinical Scores	Median (IQR)
Gender	83 males (61.5%)–52 females (38.5%)
Age (years)	63.8 (56.0–72.0)
Heart rate (beats/min)	85 (75–95)
Respiratory rate (breaths/min) ^§^	21 (18–26)
SpO_2_ (%) ^§^	96 (94–98)
Temperature (°C)	36.5 (36.1–36.7)
Systolic pressure (mm Hg)	129 (120–140)
Diastolic pressure (mm Hg)	75 (70–85)
NEWS2	5 (4–6)
Days from illness onset to hospital admission	6 (4–8)
Comorbidities	
BMI ≥ 30	35
Current or former smokers	16
Charlson Comorbidity Index	2 (1–3)
Laboratory Findings	
Hemoglobin (g/dl)	14.2 (12.6–15.1)
RDW-CV (%)	13.4 (12.9–14.0)
White blood cells (cell count × 10^3^/µL)	7.0 (5.1–9.7)
Neutrophils (cell count × 10^3^/µL)	5.7 (4.2–8.6)
Lymphocytes (cell count × 10^3^/µL)	0.7 (0.5–1.0)
Platelets (cell count × 10^3^/µL)	205 (162–263)
ALT (U/L)	37 (28–55)
AST (U/L)	42 (32.0–57.0)
Bilirubin (mg/dl)	0.6 (0.5–0.8)
Creatinine (mg/dl)	0.8 (0.6–1.0)
Glomerular filtration rate (ml/min)	89 (70–103)
CRP (mg/dl)	8.3 (4.4–13.0)
LDH (U/L)	718 (554–873)
Erythrocyte sedimentation rate (mm/h)	40 (26–53)
Troponin I (ng/mL)	7 (3–15)
Ferritin (ng/mL)	830.0 (410.0–1347.5)
D-dimer (µg/L)	721 (517–1317)
Albumin (g/dl)	4.0 (3.7–4.2)
IL-6 (pg/mL)	10.5 (4.3–27.7)
Arterial Blood Gas Test ^§^	
pO_2_ (mm Hg)	70.0 (59.5–80.5)
pH	7.5 (7.4–7.5)
pCO_2_ (mm Hg)	36.5 (33.0–39.0)
PiO_2_/FiO_2_	146 (119–180)

**Table 2:** Common symptoms upon hospital admission.

Symptoms	Frequency (%)
Dyspnea	63.0
Dry cough	38.5
Asthenia	28.1
Productive cough	11.1
Myalgia	11.1
Headache	7.4
Thoracic pain	5.2

**Table 3:** Comparison of plasma CGRP levels (ng/mL) at baseline (t0) and after 7 days (t7) of hospitalization between patients with negative disease evolution (in-hospital death or ICU admission) vs. all other patients. Values are expressed as median (IQR). Bold text highlights the statistically significant results. N = number of analyzed patients.

	Negative Disease Evolution	All Other Patients	Z	*p*-Value
t0	(N = 29) 1.02 [0.83–1.42]	(N = 106) 0.91 [0.71–1.20]	1.9129	0.05
t7	(N = 11) 1.20 [0.90–1.40]	(N = 55) 1.20 [0.80–1.70]	−0.3532	0.72

**Table 4:** Comparison of plasma CGRP levels (ng/mL) at baseline (t0) and after 7 (t7) days of hospitalization between patients with a faster recovery (discharge and/or NEWS2 ≤ 2 for at least 24 h within 14 days of hospitalization) vs. all other patients. Values are expressed as median (IQR). Bold text highlights the statistically significant results. N = number of analyzed patients.

	Faster Clinical Recovery	All Other Patients	Z	*p*-Value
t0	(N = 87) 0.90 [0.70–1.12]	(N = 48) 0.97 [0.80–1.40]	−2.2481	0.02
t7	(N = 45) 1.12 [0.80–1.50]	(N = 21) 1.27 [1.10–1.68]	−0.7238	0.47

**Table 5:** Comparison of plasma CGRP levels (ng/mL) at baseline and after 7 days of hospitalization between patients with or without pulmonary intravascular coagulopathy. Values are expressed as median (IQR). Bold text highlights the statistically significant results. N = number of analyzed patients.

	No Pulmonary Intravascular Coagulopathy	Pulmonary Intravascular Coagulopathy	Z	*p*-Value
t0	(N = 74) 0.88 [0.70–1.08]	(N = 61) 0.99 [0.78–1.38]	−2.6641	<0.01
t7	(N = 35) 1.10 [0.70–1.86]	(N = 31) 1.20 [0.90–1.50]	−0.3667	0.71

**Table 6:** Multivariate stepwise logistic regression of plasma CGRP levels at baseline (t0), predicting a negative disease evolution (in-hospital death or ICU admission) including demographic and clinical severity variables. The variables entered in the model are reported in the table. NEWS2 score and PiO_2_/FiO_2_ did not enter the model.

Predictors	Coefficient	Standard Error	*p*-Value	Odds Ratio	95% Confidence Interval
CGRP (ng/mL)	1.0436	0.496	0.04	2.84	1.07–7.51
Age	0.0758	0.023	<0.01	1.08	1.03–1.13
Sex (female)	−1.7037	0.570	<0.01	0.18	0.06–0.56

**Table 7:** Multivariate stepwise logistic regression of plasma CGRP concentration at baseline (t0) predicting a faster clinical recovery (discharge and/or NEWS2 ≤ 2 for at least 24 h within 14 days of hospitalization), including demographic and disease severity variables. The variables that entered the model are reported in the table. The gender and NEWS2 score did not enter the model.

Predictors	Coefficient	Standard Error	*p*-Value	Odds Ratio	95% Confidence Interval
CGRP (ng/mL)	−0.9738	0.496	0.05	0.38	0.14–1.00
Age	−0.0745	0.020	<0.01	0.93	0.89–0.96
PiO_2_/FiO_2_	0.0122	0.004	<0.01	1.01	1.00–1.02

**Table 8:** Multivariate stepwise logistic regression of plasma CGRP levels at baseline (t0) predicting the development of pulmonary vascular complications, including demographic and disease severity variables. The variables entered in the model are reported in the table. Age, gender, and NEWS2 score did not enter the model.

Predictors	Coefficient	Standard Error	*p*-Value	Odds Ratio	95% Confidence Interval
CGRP (ng/mL)	1.0726	0.458	0.02	2.92	1.19–7.17
PiO_2_/FiO_2_	−0.0079	0.003	0.01	0.99	0.99–1.00

**Table 9:** Multiple correlation analyses between baseline (t0) plasma CGRP levels (ng/mL) and laboratory parameters. Bold text highlights the statistically significant results.

Laboratory Parameters (Determined as)	Correlation Coefficient (CGRP vs Lab. Parameter)	*p*-Value
Hemoglobin (g/dL)	0.1810	0.472
RDW-CV (%)	0.1300	0.607
White blood cells (cell count × 10^3^/µL)	0.1532	0.544
Neutrophils (cell count × 10^3^/µL)	0.1975	0.432
Eosinophils (cell count × 10^3^/µL)	−0.0793	0.754
Lymphocytes (cell count × 10^3^/µL)	−0.1434	0.570
Platelets (cell count × 10^3^/µL)	−0.3182	0.198
ALT (U/L)	0.1036	0.682
AST (U/L)	0.2352	0.348
Bilirubin (mg/dL)	−0.0081	0.974
Creatinine (mg/dL)	0.2382	0.341
Glomerular filtration rate (mL/min)	−0.4020	0.098
CRP (mg/dl)	0.3211	0.194
LDH (U/L)	−0.1573	0.533
Erythrocyte sedimentation rate (mm/h)	−0.1291	0.610
Troponin I (ng/mL)	0.0379	0.881
Ferritin (ng/mL)	−0.1363	0.590
D-dimer (µg/L)	0.1117	0.659
Albumin (g/dL)	−0.2734	0.272
Neutrophils/Lymphocytes ratio	0.3697	0.131
**IP-10 (pg/mL)**	**0.4852**	**0.041**
Eotaxin (pg/mL)	0.0028	0.991
FGF (pg/mL)	−0.1074	0.671
G-CSF (pg/mL)	0.1736	0.491
GM-CSF (pg/mL)	−0.1761	0.485
IFN-γ (pg/mL)	0.0641	0.800
IL-1 (pg/mL)	−0.4257	0.078
IL-1 Ra (pg/mL)	0.0575	0.821
IL-2 (pg/mL)	0.2442	0.329
IL-4 (pg/mL)	−0.2733	0.273
IL-5 (pg/mL)	−0.0227	0.929
IL-6 (pg/mL)	0.0851	0.737
IL-7 (pg/mL)	0.0041	0.987
IL-8 (pg/mL)	0.1196	0.636
IL-9 (pg/mL)	−0.1222	0.629
IL-10 (pg/L)	0.1790	0.477
IL-12 (pg/mL)	0.1415	0.576
IL-13 (pg/mL)	0.1689	0.503
IL-15 (pg/mL)	−0.0008	0.998
IL-17 (pg/mL)	0.3456	0.160
MCP-1 (pg/mL)	−0.0982	0.698
MIP-1α (pg/mL)	0.1384	0.584
MIP-1β (pg/mL)	−0.0889	0.726
PDGF (pg/mL)	−0.0986	0.697
RANTES (pg/mL)	−0.0728	0.774
TNF-α (pg/mL)	−0.2438	0.330
VEGF (pg/mL)	−0.2673	0.283

## Data Availability

The original contributions presented in the study are included in the article. Further inquiries can be directed to the corresponding author.
